# Timing major conflict between mitochondrial and nuclear genes in species relationships of *Polygonia *butterflies (Nymphalidae: Nymphalini)

**DOI:** 10.1186/1471-2148-9-92

**Published:** 2009-05-07

**Authors:** Niklas Wahlberg, Elisabet Weingartner, Andrew D Warren, Sören Nylin

**Affiliations:** 1 Laboratory of Genetics, Department of Biology, University of Turku, 20014 Turku, Finland; 2 Department of Zoology, Stockholm University, 106 91 Stockholm, Sweden; 3 McGuire Center for Lepidoptera and Biodiversity, Florida Museum of Natural History, University of Florida, SW 34th Street and Hull Road, PO Box 112710, Gainesville, FL 32611-2710, USA; 4 Museo de Zoología, Departamento de Biología Evolutiva, Facultad de Ciencias, Universidad Nacional Autónoma de México, Apdo. Postal 70-399, México, DF 04510 México

## Abstract

**Background:**

Major conflict between mitochondrial and nuclear genes in estimating species relationships is an increasingly common finding in animals. Usually this is attributed to incomplete lineage sorting, but recently the possibility has been raised that hybridization is important in generating such phylogenetic patterns. Just how widespread ancient and/or recent hybridization is in animals and how it affects estimates of species relationships is still not well-known.

**Results:**

We investigate the species relationships and their evolutionary history over time in the genus *Polygonia *using DNA sequences from two mitochondrial gene regions (COI and ND1, total 1931 bp) and four nuclear gene regions (EF-1α, wingless, GAPDH and RpS5, total 2948 bp). We found clear, strongly supported conflict between mitochondrial and nuclear DNA sequences in estimating species relationships in the genus *Polygonia*. Nodes at which there was no conflict tended to have diverged at the same time when analyzed separately, while nodes at which conflict was present diverged at different times. We find that two species create most of the conflict, and attribute the conflict found in *Polygonia satyrus *to ancient hybridization and conflict found in *Polygonia oreas *to recent or ongoing hybridization. In both examples, the nuclear gene regions tended to give the phylogenetic relationships of the species supported by morphology and biology.

**Conclusion:**

Studies inferring species-level relationships using molecular data should never be based on a single locus. Here we show that the phylogenetic hypothesis generated using mitochondrial DNA gives a very different interpretation of the evolutionary history of *Polygonia *species compared to that generated from nuclear DNA. We show that possible cases of hybridization in *Polygonia *are not limited to sister species, but may be inferred further back in time. Furthermore, we provide more evidence that Haldane's effect might not be as strong a process in preventing hybridization in butterflies as has been previously thought.

## Background

Phylogenetics at the species-level is becoming increasingly important in the study of processes underlying speciation [1,2]. Most species-level phylogenies have until recently been based on only mitochondrial DNA (mtDNA) due to the ease of PCR amplification and its perceived suitability, e.g. due to maternal inheritance (shorter time for coalescence than nuclear DNA (nDNA) because of smaller N_e_), lack of recombination and relatively high mutation rate. However, species phylogenies are not necessarily the same as gene phylogenies [3,4], as different genes might have different histories. Genes involved with speciation, affecting such traits as hybrid incompatibility, as well as sex chromosomes should be more differentiated between species and less likely to introgress than autosomes [5-8]. Different processes such as random sorting of homoplasy, ancient polymorphism and hybridization with gene introgression can obscure patterns of species relationships. Information from different regions of genomes such as mitochondrial DNA, nuclear DNA (from sex chromosomes as well as from autosomes) and microsatellites are thus necessary in investigating the evolutionary history of a group of closely related species.

As species-level molecular phylogenies based on both mitochondrial and nuclear markers have become more common, it has become clear that there is often well-supported conflict between the genomes for certain clades in given phylogenies [9]. Recent work is pointing to major conflict between mtDNA and nuclear DNA in species-level phylogenetic analyses [6,9-15]. The conflict is often attributed to ancient or recent hybridization [9,12-14] or incomplete lineage sorting [15]. Hybridization, a well-accepted process in plants, appears to be more common also among closely related animals than previously thought [16]. Kronforst [17] showed in *Heliconius *butterflies that gene flow between species can proceed for long periods of time after divergence.

Although phylogenies give us a hypothesis of species relationships, they tell us little about the processes involved in diversification on their own. More information is needed to discover reasons for diversification, such as geographic location of specimens used and times of divergence of lineages. Contemporary sympatric species might have been allopatric when the two lineages diverged and without well-sampled species it is even harder to draw any conclusions about movements of species and populations. Knowledge about when divergences of lineages have happened in a given group of species may give insight to the processes behind the conflicts in phylogenetic signal. However, the temporal framework has rarely been studied for such conflicts.

Here we study the relationships of species in the genus *Polygonia *(Lepidoptera: Nymphalidae), which have been used as model taxa in numerous studies on the evolution of insect-host plant interactions [18-21], phenotypic plasticity in life-history traits [22,23], effects of environment on distribution [24] and insect physiology [25,26]. *Polygonia *is a genus thought to include five Palaearctic species (*P. c-album, P. c-aureum, P. egea, P. gigantea *and *P. interposita*), and nine Nearctic species, seven in the United States and Canada (*P. comma, P. faunus, P. gracilis, P. interrogationis, P. oreas, P. progne *and *P. satyrus *[27,28]) and two endemic to Mexico (*P. g-argenteum *and *P. haroldii*). The taxonomic status of some of these species is disputed. *Polygonia interposita *has been treated as a subspecies of *P. c-album *[29] but was suggested to be a species-level taxon by Churkin [30]. So far, this taxon has not been included in any earlier molecular studies. We have tentatively treated *P. zephyrus *as a species separate from *P. gracilis *[following [31]] although this status is unclear; *P. zephyrus *is often considered conspecific with *P. gracilis *[32]. These two taxa (*P. zephyrus and P. gracilis*) are morphologically distinguishable at the extremes of their ranges but between those "ends of a cline" a broad zone exists where intermediate forms occur (ADW pers. obs.). This may be an example of incipient speciation or secondary contact between two species. Earlier, *P. oreas *was sometimes considered a subspecies of *P. progne*, but in a recent study [33], *P. oreas *was found to be closely related to *P. gracilis*.

According to previous analyses, the ancestor of *Polygonia *was distributed in the Palaearctic and there have been two colonization events into the Nearctic region [33]. The ancestral host plants were most likely "urticalean rosids" (which includes the closely related plant families Urticaceae, Ulmaceae, Cannabaceae and Celtidaceae) [21,34]. Many *Polygonia *species are still restricted to plants from this group but some species have included additional or shifted completely to other plant families, such as Betulaceae, Ericaceae, Grossulariaceae and Salicaceae. In previous studies [21,34] phylogenetic trees have been used to infer ancestral host plant ranges used by butterflies in the subfamily Nymphalinae. The results imply that when host plant range has expanded, an increase in the rate of net diversification has followed. In order to understand in more detail the dynamics of host plant use and diversification in the *Polygonia *butterflies in particular, and insects in general, it is necessary to generate a hypothesis of the evolutionary history of the group.

Species of *Polygonia *have been included in several earlier phylogenetic studies [33,35,36], but the relationships of some species have not been stable and conflict between datasets has been noted [36]. In this study, we present a hypothesis of the evolution of this genus in which all currently accepted species and most subspecies have been included. We then apply a temporal framework in order to illuminate the causes of major conflicts between genomic datasets.

## Results

**Figure 1:**
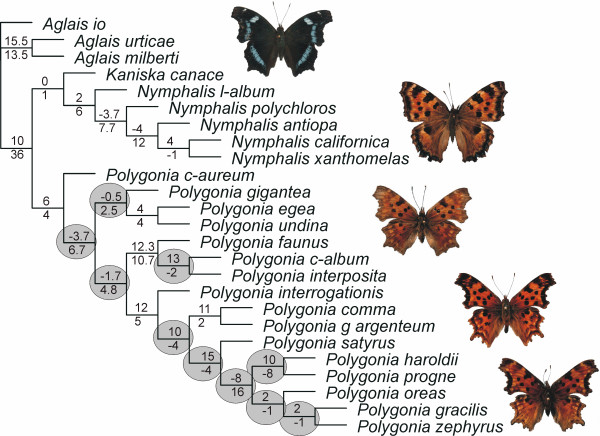
**Combined analysis of all genes**. Values above branches are the Partitioned Bremer Support (PBS) values for the combined mitochondrial gene partition and values below branches are the PBS values for the combined nuclear gene partition. Grey circles highlight nodes with strong conflict within the *Polygonia *clade. Pictured butterflies are from top to bottom *Kaniska canace*, *Nymphalis polychloros*, *Polygonia c-album*, *Polygonia satyrus *and *Polygonia zephyrus*.

**Figure 2:**
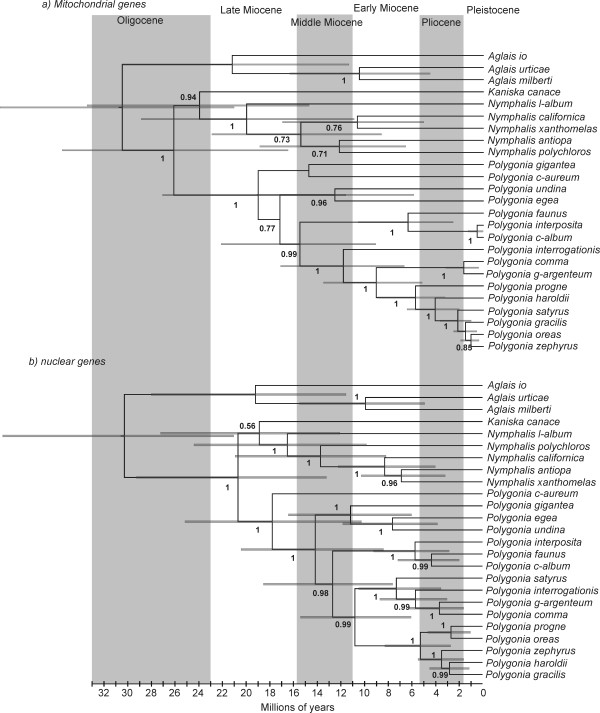
**Dated phylogenies of *Polygonia***. a) based on mtDNA b) based on nDNA. Values below the branches are posterior probabilities for the nodes to the right of the numbers.

Combined analysis with Partitioned Bremer Support (PBS) of the 25-taxon dataset showed that there was strong conflict between the mitochondrial partition and the nuclear partition at almost all nodes within the genus *Polygonia *(Figure 1). Analysis of the two genomic datasets separately showed that the topology was different at these conflicting nodes (Figure 2). Despite the conflict, there are several well-supported clades at which there is no conflict between datasets. The genus *Polygonia *without the species *Kaniska canace *is strongly supported by all datasets. The position of *K. canace *as sister to the genus *Nymphalis *is not well-supported in the cladistic analyses, but is supported by all Bayesian analyses of combined and separate data. The sister species relationships of *P. egea *and *P. undina*, as well as *P. comma *and *P. g-argenteum *are well supported. The clade containing *P. c-album*, *P. interposita *and *P. faunus*, as well as the clade containing all the rest of the Nearctic species (except *P. faunus*), are also well-supported.

The level of conflict between the different genomic datasets is particularly evident when comparing the separate analyses, where species relationships are quite different with relatively good support (Figure 2). These topologies were not dependent on method used for analysis, and thus the phylogenetic signal found within the mitochondrial and nuclear datasets appears to be strong. The exception is *P. gigantea*, which receives strong support as the sister to *P. undina+P. egea *with the nuclear dataset, but little or no support as sister to *P. c-aureum *with the mitochondrial dataset (Figure 2). Interestingly, the estimated times of divergence for clades which are common to the two datasets are similar regardless of which dataset one uses (with the caveat that the confidence intervals are very wide). Thus the *Polygonia *clade is estimated to have started diverging 18–19 million years ago (mya), the *P. c-album *to Nearctic *Polygonia *clade between 13 and 16 mya, the Nearctic *Polygonia *at 11–12 mya and the *P. progne *to *P. gracilis *clade between 5 and 6 mya (Figure 2).

Of particular interest in the separate analysis of the mitochondrial and nuclear datasets is the position of *P. interposita *as sister to *P. c-album *(indeed with almost identical COI haplotypes) based on mtDNA, but as sister to *P. c-album+P. faunus *based on nDNA. Also the position of *P. satyrus *as very closely related to *P. gracilis+P. oreas+P. zephyrus *based on mtDNA, but as sister to *P. interrogationis+P. g-argenteum+P. comma *based on nDNA. Finally, the position of *P. oreas *as part of the *P. gracilis+P. zephyrus *clade based on mtDNA, but as sister to *P. progne *based on nDNA (Figure 2).

**Figure 3:**
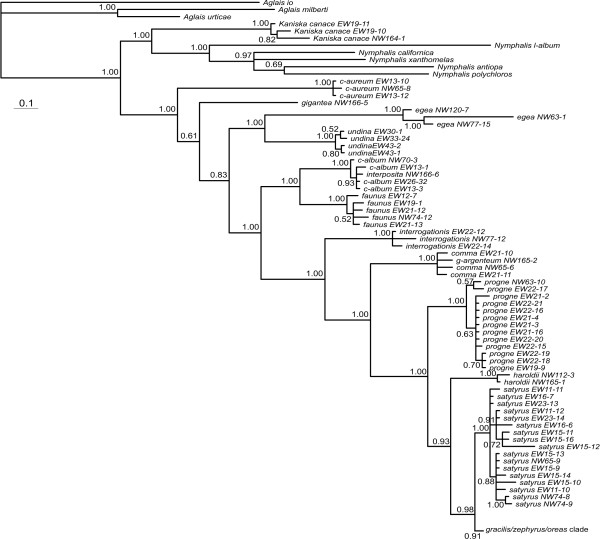
**Topology of haplotypes from Bayesian analysis based on the mtDNA COI**. Details of *gracilis*/*zephyrus*/*oreas *clade are shown in the haplotype network in Figure 8a. Values below the branches are posterior probabilities for the nodes to the right of the numbers.

Increasing the sample size for each gene region and analyzing them separately brings some light to these patterns. The COI tends to have very little variation within species, but substantial variation between species (Figure 3). The exceptions are *P. interposita*, which is almost identical to *P. c-album*; *P. g-argenteum*, which is very similar to *P. comma*; and *P. gracilis*, *P. zephyrus *and *P. oreas*, which are all very similar to each other even to the point of sharing haplotypes between the three taxa. The position of *P. satyrus *is consistent with the 25-taxon dataset, and shows some variation within the species.

**Figure 4:**
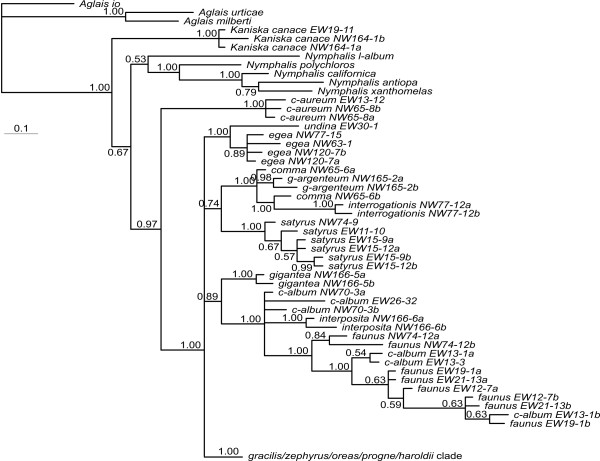
**Topology of haplotypes from Bayesian analysis based on the nDNA EF-1α**. Details of *gracilis*/*zephyrus*/*oreas*/*progne*/*haroldii *clade are shown in the haplotype network in Figure 8b. Values below the branches are posterior probabilities for the nodes to the right of the numbers.

**Figure 5:**
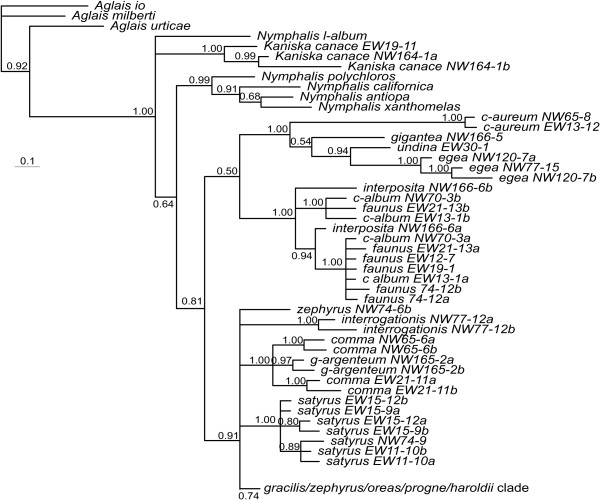
**Topology of haplotypes from Bayesian analysis based on the nDNA GAPDH**. Details of the *gracilis*/*zephyrus*/*oreas*/*progne*/*haroldii *clade are shown in the haplotype network in Figure 8c. Values below the branches are posterior probabilities for the nodes to the right of the numbers.

**Figure 6:**
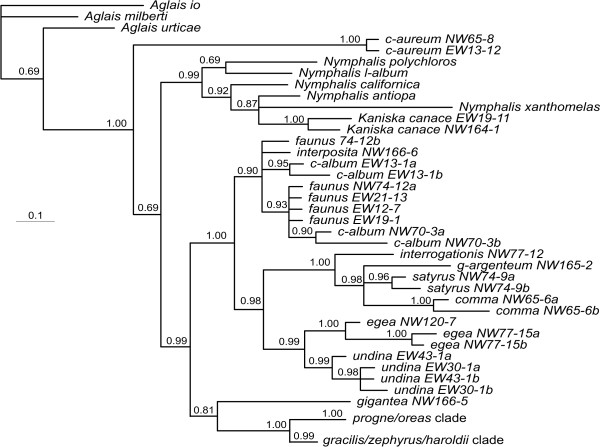
**Topology of haplotypes from Bayesian analysis based on the nDNA RpS5**. Details of the *progne*/*oreas *clade and the *gracilis*/*zephyrus*/*haroldii *clade are shown in the haplotype network Figure 8d. Values below the branches are posterior probabilities for the nodes to the right of the numbers.

**Figure 7:**
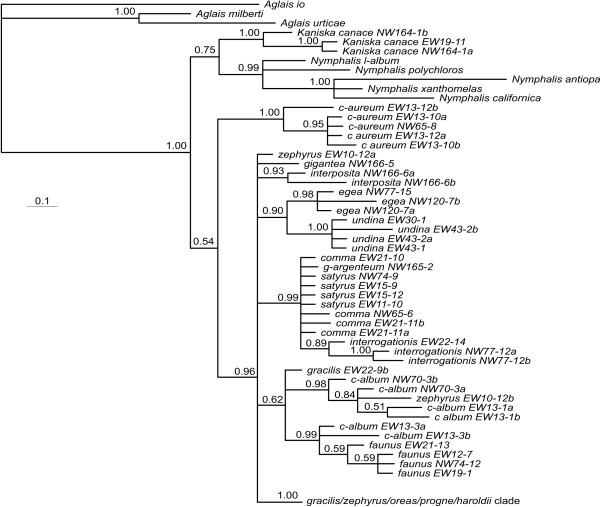
**Topology of haplotypes from Bayesian analysis based on the nDNA wgl**. Details of the *gracilis*/*zephyrus*/*oreas*/*progne*/*haroldii *clade are shown in the haplotype network Figure 8e. Values below the branches are posterior probabilities for the nodes to the right of the numbers.

**Figure 8:**
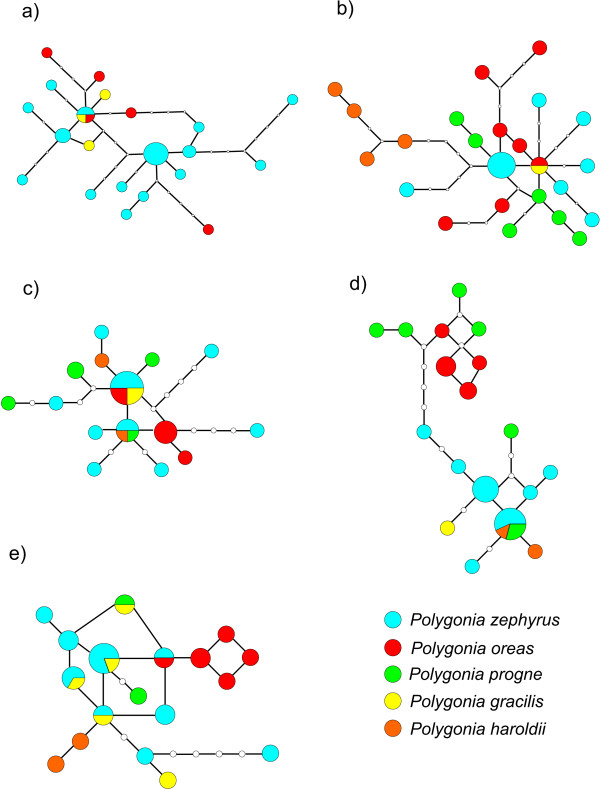
**Minimum spanning networks**. a) COI b) EF-1α c) GAPDH d) RpS5 and e) wgl. Size of the circles are directly proportional to the number of individuals with that haplotype. Small white circles indicate a missing haplotype. Each branch is equivalent to one basepair change. Circles with more than one pattern show the proportion of each species. The colour coding are as follows; blue – *P. zephyrus*, red – *P. oreas*, green – *P. progne*, yellow – *P. gracilis *and orange – *P. haroldii*.

The nuclear gene regions show quite different topologies when analyzed on their own, but several patterns are consistent between them (Figure 4, Figure 5, Figure 6, Figure 7 and Figure 8). First of all, the haplotypes of GAPDH and wgl are very similar in the taxa *P. gracilis*, *P. zephyrus*, *P. haroldii*, *P. oreas *and *P. progne *(Figure 5, Figure 7 and Figure 8). The haplotypes of EF-1α, RpS5 and wgl in *P. satyrus *are more related to *P. comma*, *P. g-argenteum *and *P. interrogationis *than to the other Nearctic *Polygonia *(Figure 4, Figure 6 and Figure 7). The nDNA haplotypes found in *P. interposita *tend to not be especially close to *P. c-album *(Figure 4, Figure 5, Figure 6 and Figure 7). Interestingly, the haplotypes of RpS5 found in *P. oreas *are very closely related to those found in *P. progne *(Figure 6 and Figure 8d), while all other nDNA haplotypes are ambiguous about this relationship. Finally, the subspecies *P. e. undina *is differentiated from *P. egea *for all sequenced genes.

Regarding the haplotype networks, we have focused on the unresolved clade of *P. zephyrus, P. gracilis *and *P. oreas *(in COI) as well as the relationships between these species and *P. progne *and *P. haroldii *(in RpS5, EF-1α, GAPDH and wgl) (Figure 8). None of the haplotype networks showed a star-like pattern, i.e., a "central" commonly shared haplotype from which other haplotypes deviate by only a few mutational steps, indicative of a rapid and recent diversification [37]. Most haplotypes were only represented by one individual. A few haplotypes were however shared, even between different species. For instance, *Polygonia oreas *shared the same haplotype with *P. gracilis *and *P. zephyrus *in the COI dataset (Figure 8a). In the EF-1α dataset one *P. oreas *haplotype is shared with *P. gracilis *(Figure 8b). Shared haplotypes were found for *P. haroldii, P. progne *and *P. zephyrus *in the GAPDH dataset as well as for *P. progne, P. zephyrus *and *P. gracilis *(Figure 8c). In the RpS5 dataset *P. haroldii*, *P. zephyrus *and *P. gracilis *shared the same haplotype (Figure 8d). Three haplotypes were shared between *P. gracilis *and *P. zephyrus*, one haplotype was shared between *P. progne *and *P. gracilis *and one haplotype was shared between *P. oreas *and *P. zephyrus *in the wgl dataset (Figure 8e). In addition, in the wgl dataset one haplotype is shared between *P. comma, P. g-argenteum *and all *P. satyrus*. However, the *P. g-argenteum *individual had many positions of missing data.

## Discussion

### Major clades in the phylogeny

Unlinked genes are expected to have independent genealogical histories [3,38], and thus combining data may not always be informative of species relationships [39]. In this study we have found that gene regions from different genomes (mtDNA and nDNA) give rather different estimates of species relationships. The phylogenetic positions of four taxa in particular need explanation: *P. satyrus*, *P. oreas, P. haroldii *and *P. interposita*. Each of these is strongly supported in different positions depending on which dataset is analysed. Before we discuss these four anomalous taxa, we will discuss the general findings for the other species, as this is the most complete study of *Polygonia *phylogeny to date, including several taxa that have never been part of a phylogenetic systematic investigation.

Previous studies have shown conflicting results on the position of the taxon "*canace*" [33,35,36], often placed in the monotypic genus *Kaniska*, but suggested to be included in *Polygonia *by Wahlberg & Nylin [36]. The present study does not corroborate that finding, but it should be noted that in contrast to the earlier study we did not include morphological data here. However, it is clear that the position of "*canace*" is not stable and it's sister relationship either to *Nymphalis *or to *Polygonia *is weakly supported. In such a case, we feel it is best to retain it in the monotypic genus *Kaniska*, in order to highlight its "oddity" and long history of independent evolution. This is of course only valid if one accepts the validity of the genera *Polygonia *and *Nymphalis*, which some consider to be a single genus *Nymphalis*, along with our *Aglais *[e.g., [40,41]]. For reasons explained in Wahlberg & Nylin [36], we feel that the genus *Polygonia *should be retained, and thus we suggest that the taxon "*canace*" be retained in the genus *Kaniska*, as is frequently done in the literature [e.g., [29], e.g., [42,43]].

There are several independent lineages within *Polygonia*. The type species of the genus, *Polygonia c-aureum*, is the sister to the rest of *Polygonia*, as has been found in previous studies [33,35,36]. *Polygonia gigantea*, included here for the first time in a phylogenetic study, is an independent lineage that is most likely sister to the *P. egea+P. undina *clade, based on the well-supported result of the nDNA dataset and the ambiguous result of the mtDNA dataset. *Polygonia undina *has mainly been considered to be a subspecies of *P. egea*, but our results show that it is genetically very distinct and the common ancestor of the two diverged as early as between 8–13 mya (Figure 2). This makes the pair older than several other species pairs in *Polygonia*, and we found no evidence of interbreeding (all genes were clearly diverged for this pair of species). We thus elevate *P. undina *to the species level (**stat. nov**.).

The clade containing *P. c-album*, *P. interposita *and *P. faunus *is well-supported and quite clearly the sister to the Nearctic clade. The interrelationships of these three species will be discussed in more detail below. The Nearctic clade including *P. satyrus*, *P. interrogationis*, *P. comma*, *P. g-argenteum*, *P. progne*, *P. oreas*, *P. haroldii*, *P. gracilis *and *P. zephyrus*, is also well-supported. Within this clade, *P. g-argenteum *(here included for the first time in a phylogenetic analysis) is clearly the sister species to *P. comma*, and apparently these two have diverged relatively recently (2–4 mya). The position of *P. interrogationis *with regard to these two species is different with the two genomic datasets. Mitochondrial DNA suggests that it is sister to the rest of the Nearctic species, while nDNA suggests that it is sister to *P. comma+P. g-argenteum*. The latter sister relationship is in fact suggested by morphological data as well [35], giving more weight to this hypothesis of phylogeny.

Our data suggest that *P. comma *and *P. g-argenteum *have diverged in the past 2–3 mya, during which time there has been considerable morphological diversification between them. Adults of *g-argenteum *are among the largest of *Polygonia *(generally the same size as *P. interrogationis*), and they lack the seasonal polyphenism (expression of dark "summer" forms) seen in *P. comma *and *P. interrogationis*. As a result, size excluded, adults of *P. satyrus *and *P. g-argenteum *share a very similar superficial resemblance (especially in the dorsal view), while adults of *P. comma *(especially dark forms) and *P. g-argenteum *appear quite different at first glance.

As an aside, it is interesting to note that apparently similar patterns of differences between mitochondrial and nuclear DNA are found in the genus *Nymphalis *(Figure 2). This warrants a separate study to see if similar forces have acted on the sister group of *Polygonia*.

### Ancient mitochondrial introgression in *P. satyrus*

Mitochondrial DNA suggests that *P. satyrus *is closely related to *P. gracilis*, *P. zephyrus *and *P. oreas*, whereas nDNA suggests very strongly that *P. satyrus *is sister to *P. interrogationis*, *P. comma *and *P. g-argenteum *(Figure 2). Morphological and ecological features, however, suggest that *P. satyrus *is more related to the latter clade. In addition to great overall phenotypic similarity between the adults and immatures of *P. satyrus *and *P. comma*, larvae of those two taxa, as well as those of *P. interrogationis *and *P. g-argenteum *[see [44]], feed on Ulmaceae, Moraceae and Urticaceae as larvae, and late-instar larvae of *P. satyrus *and *P. comma *make very similar larval nests out of altered host plant leaves [45]. *Polygonia satyrus *is largely parapatric with respect to *P. comma*, as the two fly in sympatry only in a limited portion of northeastern North America, and rarely in eastern Colorado, where *P. comma *is present only as uncommon vagrant individuals from the east [46].

According to our estimates of times of divergence (Figure 2), *P. satyrus *diverged from the ancestral populations between 7–8 mya based on nDNA, whereas the result from mtDNA suggests that the divergence happened much more recently, about 2 mya. Given that the nDNA estimate of divergence time is older than that from mtDNA, it is possible that the presence of an "alien" mtDNA lineage in *P. satyrus *may be the result of ancient introgression from the ancestor of *P. gracilis+P. zephyrus+P. haroldii*, which could have happened some 2–3 mya (prior to the onset of the Pleistocene glacial periods). The current sympatric distribution of *P. satyrus *vs. *P. gracilis*+*P. zephyrus *(the geographic distribution of these taxa is almost identical) highlights the potential for gene exchange in the recent past and present. Given also that all mtDNA haplotypes found to date in *P. satyrus *are very similar, yet all nDNA haplotypes are more related to the *P. interrogationis *clade, it is possible that repeated population bottlenecks during the glacial cycles have wiped out the original mtDNA lineages from *P. satyrus*, by chance leaving the current introgressed lineage in extant populations. Lack of gene flow during the last 2 million years has now resulted in reciprocal monophyly to evolve in *P. satyrus *and *P. gracilis+P. zephyrus*+*P. haroldii*. Such a speculative scenario could be corroborated by more extensive sampling of *P. satyrus *populations across North America, which could make possible coalescense modeling to rule out any possibility that the conflicting results can be explained by ancient polymorphisms [39,47].

In butterflies females are the heterogametic sex and it is accepted that "Haldane's rule" [48] is an important phenomenon, ie. introgression of the maternally inherited mtDNA will not enter the new gene pool due to low viability or sterility of female F_1 _offspring [see [49]]. Presgraves [50] showed that hybrid sterility and inviability are common in Lepidoptera and evolve gradually. In those studies of butterflies where both mtDNA and nDNA have been screened, introgression in nDNA but not mtDNA has been found between *Papilio machaon *and *P. hospiton *(Papilionidae) [7] as well as between *Heliconius cydno *and *H. melpone *(Nymphalidae) [13,14]. However, mtDNA introgression has been found between the latter species pair in another study [8], suggesting that the wide acceptance of Haldane's rule needs to be questioned. In the case of *Polygonia satyrus *we have no knowledge of whether hybrid female offspring are sterile or not, but even if hybrids between contemporary *P. satyrus *and species from the *P. gracilis *clade are inviable this may not have been the case when (if) introgression occured.

### Recent mitochondrial introgression in *P. oreas*

The mtDNA haplotypes found in *P. oreas *are very similar to those found in *P. gracilis *and *P. zephyrus*, and one haplotype is shared between these species. In the nDNA datasets, haplotypes are shared between *P. oreas, P. gracilis *and *P. zephyrus *for EF-1α but not for the other genes, and in the case of RpS5, haplotypes of *P. oreas *are clearly more related to *P. progne *(Figure 4, Figure 5, Figure 6, Figure 7, and Figure 8e). *Polygonia oreas *has been considered a subspecies of *P. progne *by various authors [e.g., [51]], thus once again, the nDNA dataset corroborates the morphological proposals of previous authors. Interestingly, both the mtDNA and the nDNA datasets suggest that the clade including the five taxa *P. progne*, *P. oreas*, *P. haroldii*, *P. gracilis *and *P. zephyrus *began diverging about 5 mya at the end of the Miocene. Based on nDNA, *P. oreas *and *P. progne *began diverging about 3 mya, whereas the divergence of *P. oreas *mtDNA is more recent. As with *P. satyrus*, no *P. oreas *COI haplotypes were found to be more related to its probable sister species *P. progne*, and it may be that bottlenecks have wiped out the original mtDNA lineages, while current introgression is introducing new genetic material into *P. oreas *from the *P. gracilis *complex (most likely from western *P. zephyrus*). On the other hand, we have sampled only 5 individuals of *P. oreas*, and it may be that a denser sampling would reveal mtDNA lineages closer to *P. progne*. *Polygonia oreas *flies in sympatry and synchrony with *P. zephyrus *throughout the vast majority of its range, the latter usually being much more abundant locally and regionally; thus there are ample opportunities for ongoing introgression between the two taxa. It should be noted that adults of some subspecies of *P. oreas*, especially *nigrozephyrus*, and some individuals of *threatfuli*, are so similar to those of sympatric *P. zephyrus *that many experienced lepidopterists cannot distinguish them (without life history information), and these two taxa were not described until 1984 and 2001, respectively (adults of these taxa are still hiding in museum series of *P. zephyrus *all over the world).

### Recent speciation of *P. haroldii *and incipient speciation of *P. gracilis/zephyrus*?

The taxa *P. haroldii*, *P. gracilis *and *P. zephyrus *appear to be related to one another in a complicated way. Mitochondrial DNA suggests that *P. haroldii *is a distinct lineage sister to the *P. gracilis/zephyrus *lineage (that includes the "alien" *P. satyrus *lineage) (Figure 3), yet the nDNA suggests that *P. haroldii *is not distinct from *P. zephyrus *(note that by chance, the *P. zephyrus *chosen for the 25-taxon analyses is rather different from the other *P. zephyrus*) (Figure 4, Figure 5, Figure 6 and Figure 7). Here the classical explanation for conflicts [52] of recent divergence with not enough time for slowly evolving nuclear genes to have segregated would appear to hold. Interestingly, *P. haroldii *(endemic to mainland Mexico) and some *P. zephyrus *(endemic to western United States and Canada, including northern Baja California, Mexico) nDNA haplotypes appear to be more related to each other, perhaps suggesting recent gene exchange between these western taxa during the Pleistocene glacial periods. Currently, the two taxa appear to be allopatrically distributed. A close relationship between *P. haroldii *and *P. zephyrus *was suggested by Krogen [53], based on morphological similarities. Beutelspacher [54] reported an unidentified species of Urticaceae as a larval foodplant for *P. haroldii *(presumably in the Valley of Mexico), although this record seems unlikely, since *P. haroldii *is usually found in immediate association with *Ribes *species (Grossulariaceae) (ADW, pers. obs.), the host plant genus utilized by *P. zephyrus*.

Our data suggest that morphological differentiation may occur rapidly in *Polygonia*, once speciation has occurred. Despite the essentially identical nDNA haplotypes between *P. zephyrus *and *P. haroldii*, the latter has diversified morphologically to the point where it cannot be confused with any other member of the genus. This was perhaps achieved through evolution of a mimetic relationship with the presumably distasteful model *Dione moneta *(Nymphalidae: Heliconiinae: Heliconiini); in flight, adults of *D. moneta *and *P. haroldii *appear nearly indistinguishable, since the metallic ventral spots of *D. moneta *frequently are not visible (ADW, pers. obs.). Currently, these two species are very often found flying in sympatry and synchrony throughout the Mexican distribution of *P. haroldii*, although the presumed model, *D. moneta*, is usually much more widespread and common than *P. haroldii*. No obvious geographic variation in morphology has been noted in *P. haroldii*.

The taxon pair *P. gracilis *and *P. zephyrus *has been treated as two hypothetically separate species in this study, but the current consensus is that these are subspecies of the same species [27,28]. Our results are ambiguous about whether these two taxa are currently diverging or merging. Morphologically, populations of far western *P. zephyrus *are separable from far eastern populations of *P. gracilis*, but there is a clear cline between the extremes, and populations found in Alberta, Canada, consist mostly of adults that cannot be confidently assigned to one or the other taxon [55]. On the one hand, our data does not distinguish between the two taxa (haplotypes are shared regardless of gene or genome inspected), but on the other hand, haplotypes are also shared with *P. progne*, *P. haroldii*, and *P. oreas*, which are distinct species-level taxa. Thus, detailed elaboration of the taxonomic status of *P. zephyrus *and *P. gracilis *will only be possible once a thorough study can be conducted, considering dozens of populations from throughout the range of the complex. The current distribution of these two taxa, with an apparently broad zone in western Canada where their identities become blurred, suggests ongoing gene flow between them, and a careful study of populations in Alberta seems warranted.

### Polygonia interposita, species or subspecies of *P. c-album*?

The rarely collected taxon *P. interposita *is found in central Asian mountains and has often been considered a subspecies of *P. c-album *[e.g., [43]]. This taxon has frequently been confused with *P. undina*, due to the somewhat similar ventral wing pattern and similar distribution. We were only able to get one specimen of *P. interposita *that gave good quality DNA. The mtDNA of this specimen was almost identical to *P. c-album *(which shows very little variation in COI across its entire range; Weingartner et al. in prep.). The nDNA, however, was quite distinct from *P. c-album*, and indeed in the 25-taxon analysis, *P. interposita *emerged as sister to *P. c-album+P. faunus*, with a divergence time estimated at about 5 mya. Could this possibly be a similar case to *P. satyrus *in North America? Only more samples of *P. interposita *would shed light on this question, but based on the current specimen, it is possible that the mitochondrial lineage of *P. c-album *has invaded the genome of *P. interposita *in recent times, resulting in a situation where the two genomes give conflicting signals regarding phylogenetic relatedness.

### Species as lineages through time

The concept of species as lineages is fast gaining support from the scientific community [56-59]. The concept takes into account that species are part of an evolutionary continuum from diverging populations to already diverged, well-defined species. Many of the multitude of proposed species concepts lie along this continuum, but are not general enough to explain the diversity we see in nature. Here we present results for a small group of well-known butterflies with a relatively stable taxonomy. Despite molecular data from 6 gene regions for a total of 4879 bp (much more than the standard in species level phylogenetic studies at the moment), we were unable to resolve the relationships of the 16 species unambiguously, mainly due to conflicts between mitochondrial and nuclear gene regions.

**Figure 9:**
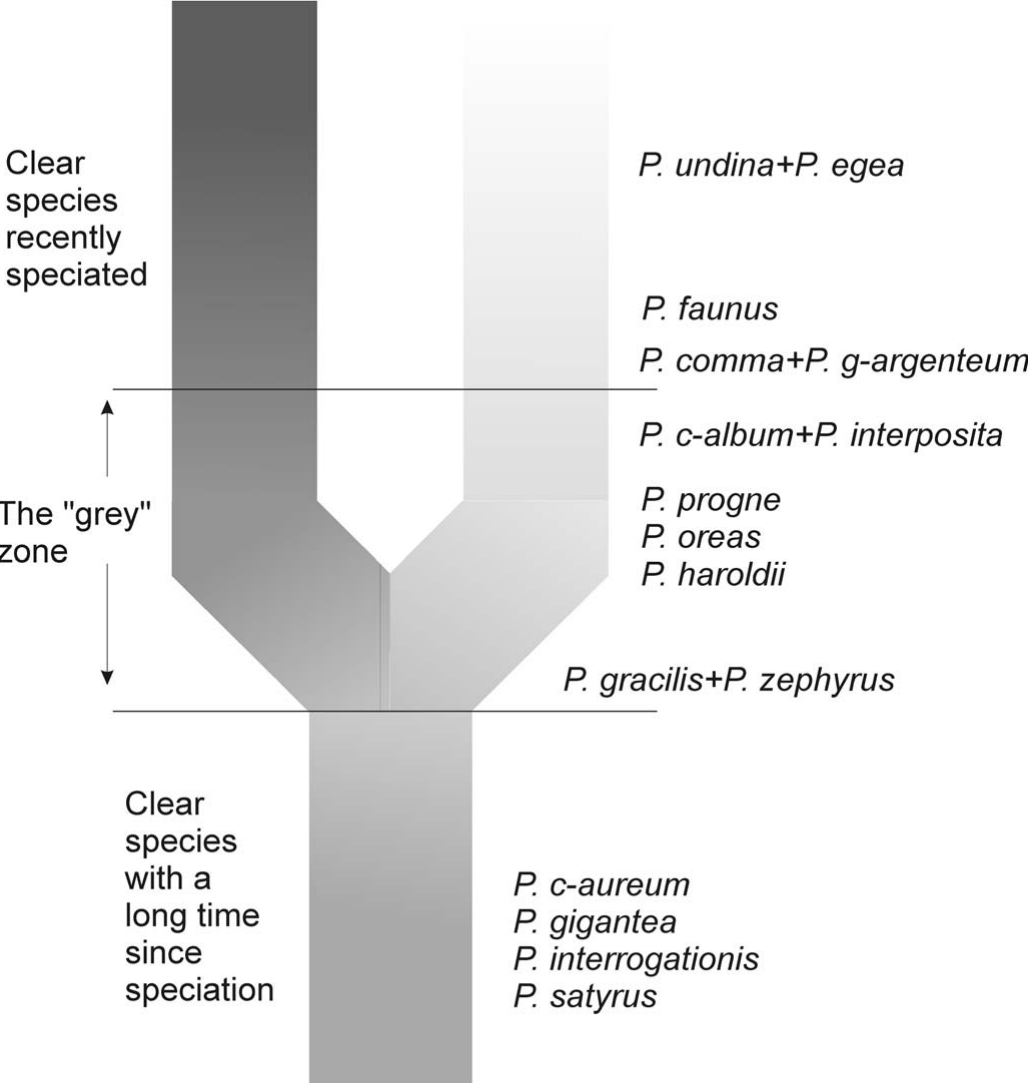
**Species through time, a summary of our results**. Species below the "grey zone" are clear independent lineages with no known closely related sister species. Species in the "grey zone" are at various stages in the speciation process. Species above the "grey zone" are closely related sister species that are separate genetic entities. They have thus by this time also become clear independent lineages ready for a future new bifurcation, should the right circumstances arise. Figure modified from de Queiroz [58].

Considering the lineage concept, it is clear that *P. c-aureum*, *P. gigantea*, *P. egea*, *P. undina*, *P. satyrus *and *P. interrogationis *have differentiated so long ago that there is no question about their taxonomic status as species (Figure 9). The species-level status of *P. comma *and *P. g-argenteum*, as well as *P. c-album*, *P. interposita *and *P. faunus *also is not really a question based on our results, but they have speciated relatively recently and in the case of *P. interposita*, may still hybridize in nature with *P. c-album*. In Figure 9, *P. faunus *is placed out of the grey zone due to the clear separation of it's populations in North America from those of *P. c-album *and *P. interposita *in Eurasia. Further down the continuum closer to the divergence events are *P. progne*, *P. oreas *and *P. haroldii *(Figure 9), which have speciated so recently that occasional gene flow may still occur between *P. oreas *and *P. progne *and/or *P. zephyrus*, but which remain taxonomic entities separate from their closest relatives. Just above the divergence line, entering into the grey zone of one or two species is the taxon pair *P. gracilis *and *P. zephyrus *(Figure 9). To really be able to say whether the two are above or below the line would require population genetic methods to see whether gene flow between the two populations is sufficiently high to consider them conspecific.

## Conclusion

In conclusion, although we now have included considerable amounts of new genetic information in an attempt to interpret the evolutionary history of *Polygonia *butterflies, we are still not able to fully understand the processes of speciation in this taxon. Especially within the Nearctic clade, more population genetic data is needed. However, our results graphically demonstrate, first, that species in this group evolve over time, sometimes over a very long time, and, second, that evidently even well-differentiated species can hybridize to the extent that different parts of their genome may suggest strongly conflicting patterns of relationships.

The results from the present study do not change the main conclusion from the study of host plant range in *Polygonia *butterflies [21]. In that paper we introduced the idea that based on the phylogeny it was possible to show that butterfly clades including species that use host plants additional to, or other than, the "urticalean rosids", included more butterfly species than the sister clade (only feeding on "urticalean rosids"). Our present results are still in agreement with the former result and there is no case in which the reverse is valid (that species restricted to "urticalean rosids" constitute more butterfly species than the sister group of species with a broader host plant range). Thus, we believe that being able to expand the host plant range will enhance speciation through colonizations and local adaptations, according to the oscillation hypothesis [60].

We have shown that the species-level relationships inferred from DNA sequence data may be strongly influenced by the markers that have been chosen. This then begs the question of how this phenomenon affects studies aimed at looking at higher levels of phylogenetic relationships, such as genera or families. Fortunately, our previous studies at higher levels have used the same markers as we have in this study [33,61-66], and results show that the COI is generally concordant with the nuclear markers at taxonomic levels above genera. This is probably due to the same stochastic processes that lead to reciprocal monophyly at the species level, i.e. given enough time, lineages (species) will go extinct, leaving sister entities that we call genera (and by default higher taxa) reciprocally monophyletic. This is not to say that all currently described genera are monophyletic entities, simply because the majority of genera have not been rigorously tested for monophyly using phylogenetic analyses.

## Methods

We sampled 96 individuals of all *Polygonia *species, as well as 8 outgroup species belonging to the genera *Nymphalis *and *Aglais *(see Table 1 and Additional File 1). Most individuals were collected by colleagues (see Acknowledgments) and sent dry to Stockholm. Total genomic DNA was extracted from two legs using QIAgen's DNEasy extraction kit, according to the manufacturer's instructions, with the exception that individuals more than 2 years old at extraction were eluted into 50 μl of elution buffer, rather than the recommended 200 μl. Voucher specimens are stored at the Department of Zoology, Stockholm University and Laboratory of Genetics, University of Turku, and can be viewed at .

**Table 1:** Summary of number of individuals per species sequenced for a given gene.

	Number of individuals sequenced					
Species	COI	ND1	wingless	EF1-α	GAPDH	RpS5

Outgroup taxa						
*Aglais io*	1	1	1	1	1	1
*Aglais milberti*	1	1	1	1	1	1
*Aglais urticae*	1	1	1	1	1	1
*Nymphalis antiopa*	1	1	1	1	1	1
*Nymphalis californica*	1	1	1	1	1	1
*Nymphalis l-album*	1	1	1	1	1	1
*Nymphalis polychloros*	1	1	1	1	1	1
*Nymphalis xanthomelas*	1	1	1	1	1	1
						
Ingroup taxa						
*Kaniska canace*	3	1	2	2	2	2
*Polygonia c-album*	4	1	3	4	2	2
*Polygonia interposita*	1	0	1	1	1	1
*Polygonia c-aureum*	3	1	3	2	2	2
*Polygonia comma*	3	1	3	1	2	1
*Polygonia egea*	3	1	2	3	2	2
*Polygonia undina*	4	0	3	1	1	2
*Polygonia faunus*	5	1	4	4	4	4
*Polygonia g-argenteum*	1	0	1	1	1	1
*Polygonia gigantea*	1	0	1	1	1	1
*Polygonia gracilis*	3	1	2	1	2	2
*Polygonia zephyrus*	24	1	8	6	8	8
*Polygonia haroldii*	2	1	2	2	2	2
*Polygonia interrogationis*	3	1	2	1	1	1
*Polygonia oreas*	5	1	4	4	4	4
*Polygonia progne*	14	1	2	3	2	2
*Polygonia satyrus*	17	1	4	4	4	1

See Additional File 1 for details of individuals, including collection locality and GenBank accession numbers.

We amplified 6 loci using PCR directly from the genomic extracts. The loci were *cytochrome oxidase subunit I *(COI) and *NADH subunit 1 *(ND1) from the mitochondrial genome, and *elongation factor-1α *(EF-1α), *wingless *(wgl), *glyceraldehyde-3-phosphate dehydrogenase *(GAPDH) and *ribosomal protein S5 *(RpS5) from different nuclear genomes. Primers and PCR protocols were taken directly from Wahlberg and Wheat [67], except for ND1, for which we followed the protocol described in Nylin et al. [35]. PCR products were cleaned using exonuclease I and calf intestine alkaline phosphatase (Fementas) and sequenced directly, using either the PCR primers or universal tails attached to the primers [for details, see [67]], on a Beckman-Coulter CEQ8000 capillary sequencer (Stockholm), or an ABI PRISMR 3130xl capillary sequencer (Turku) using dye terminator sequencing kits according to the recommendations of manufacturers.

All six genes were initially amplified for a selection of 25 taxa (8 outgroup species and 17 taxa of *Polygonia*). In order to verify patterns of strong conflict between the mitochondrial and nuclear genes [33,36], a further 77 individuals of *Polygonia *and four individuals of *Kaniska canace *were amplified and sequenced for COI, 27 individuals of *Polygonia *for EF-1α, 30 individuals of *Polygonia *for wgl, 23 individuals of *Polygonia *for GAPDH and 20 individuals of *Polygonia *for RpS5. Two individuals of *Kaniska canace *were amplified and sequenced for all nuclear genes.

Resulting chromatograms were examined by eye in BioEdit [68] and any heterozygous positions (two equally sized peaks observed at one position) were coded with IUPAC ambiguity codes. All sequences are from protein-coding genes and thus alignment was trivial. As noted in previous publications [33,35,36], a one-codon deletion was inferred in the wgl sequence of the three species of *Aglais*. Heterozygous sequences were separated manually into haplotypes. For sequences with only one heterozygous position, this was trivial. For those with two or more heterozygous positions, one haplotype was assumed to be identical to a common haplotype found in other individuals of the same species. This was possible in all cases.

The previously noted strong conflict between two mitochondrial and two nuclear genes [33,36] was investigated with a total evidence approach and Partitioned Bremer Support (PBS) on the 25-taxon dataset. Results suggested that mitochondrial and nuclear partitions continued to conflict with the addition of new nuclear gene regions. We thus analysed the combined mitochondrial genes and the combined nuclear genes to obtain estimates of relationships based on the mitochondrial genome and the nuclear genome, respectively. The two genome sets were analysed separately, but combined within each set (ie. COI+ND1 and EF-1α+GAPDH+RpS5+wgl) and will be referred to as the mitochondrial data and the nuclear data, respectively. Parsimony analyses were conducted using a heuristic search algorithm in the program TNT [69] on the equally weighted data set. The data were subjected to 100 random addition rounds of successive Sectorial, Ratchet, Drift and Tree Fusing searches [70-72]. We evaluated the character support for the clades in the resulting cladograms using Bremer support [73,74] and Partitioned Bremer support [75,76]. The scripting feature of TNT was used to calculate these values [see [64]].

Bayesian inference of phylogeny and times of divergence were estimated using the program BEAST v1.4.6 [77]. Both datasets were analysed under the GTR+ Γ model with a relaxed clock, allowing branch lengths to vary according to an uncorrelated Lognormal distribution [78]. The tree prior was set to the Yule process, and the "treeModel.RootHeight" prior (i.e., the age at the root of the tree) was set to 33 million years (with a standard deviation of 5 million years), in accordance with results from Wahlberg [79]. All other priors were left to the defaults in BEAST. Parameters were estimated using 2 independent runs of 1 million generations each (with a pre-run burn-in of 10000 generations), with parameters sampled every 1000 generations. Convergence was checked in the Tracer v1.4.6 program and summary trees were generated using TreeAnnotator v1.4.6, both part of the BEAST package.

To confirm that Bayesian analyses converged on the same topology, the data were also analyzed with MrBayes 3.1 [80]. The Bayesian analysis was performed on the combined data set with parameter values estimated separately for each gene region using the "unlink" command and the rate prior (ratepr) set to "variable". The analysis was run twice simultaneously for 2 million generations, with four chains (one cold and three heated) and every 500^th ^tree sampled. The first 500 sampled generations discarded as burn-in (based on a visual inspection of when log likelihood values reached stationarity), leaving 3501 sampled generations for the estimation of posterior probabilities. Results of the two simultaneous runs were compared for convergence using Tracer v1.4.6 [77].

The expanded single-gene datasets were analysed separately after separating heterozygotes into haplotypes. These datasets were analysed using both parsimony and Bayesian methods in TNT and MrBayes 3.1, respectively. Search parameters were as above, except the single datasets were not partitioned in any way.

In order to further investigate the resulting polytomies, we constructed a haplotype network in TCS [81], which shows how haplotypes are connected to each other. In this program, the gene genealogies from DNA sequences are estimated with statistical parsimony according to Templeton et al. [82]. We focused on the Nearctic *Polygonia *species (excluding *P. faunus*). Regions of missing basepairs were removed and we performed analyses of all Nearctic taxa as well as subsets of clades. The datasets are comprised of 1430 bp for COI, 1240 bp for EF-1α, 392 bp for wgl, 691 bp for GAPDH and 617 bp for RpS5.

## Authors' contributions

NW, SN and EW conceived the study, EW did most of the labwork, NW and EW carried out the analyses and wrote the manuscript. ADW helped interpret phylogenetic patterns and wrote part of the discussion. All authors partook in discussions during analysis and writing, read and approved the final manuscript.

## Supplementary Material

Additional file 1 **List of specimens sampled in this study**. Voucher codes, locality where the taxa were collected and GenBank accession numbers for genes sequenced. Photos of vouchers can be viewed at . Click here for file
