# Supplementary material for: Timing major conflict between mitochondrial and nuclear genes in species relationships of Polygonia butterflies (Nymphalidae: Nymphalini)
Source: BMC Evol Biol. 2009 May 7;9:92. doi: 10.1186/1471-2148-9-92 (PMC2688511; doi:10.1186/1471-2148-9-92)
Supplement: Additional file 1 — List of specimens sampled in this study. Voucher codes, locality where the taxa were collected and GenBank accession numbers for genes sequenced. Photos of vouchers can be viewed at . [file 1471-2148-9-92-S1.doc]

Additional File 1. List of species sampled in this study with identification numbers and locality where the taxa were collected along with information which genes sequenced. Photos of vouchers can be viewed at http://nymphalidae.utu.fi/db.php.

| Species | ID | Source of specimen | COI | ND1 | wingless | EF1-α | GAPDH | RpS5 |
| --- | --- | --- | --- | --- | --- | --- | --- | --- |
| Outgroup taxa |  |  |  |  |  |  |  |  |
| *Aglais io* | NW63-16 | Sweden | AY248785 | AF412737 | AF412766 | AY248810 | FJ639521 | FJ639576 |
| *Aglais milberti* | NW77-14 | Washington, USA | AY248787 | FJ639544 | AY248828 | AY248812 | FJ639523 | FJ639578 |
| *Aglais urticae* | NW63-3 | Sweden | AY248786 | AF412753 | AF412777 | AY248811 | FJ639522 | FJ639577 |
| *Nymphalis antiopa* | NW70-2 | Sweden | AY218246 | FJ639542 | AY218284 | AY218266 | FJ639524 | FJ639579 |
| *Nymphalis californica* | NW74-14 | Oregon, USA | AY248789 | FJ639543 | AY248830 | AY248814 | FJ639525 | FJ639580 |
| *Nymphalis l-album* | NW78-1 | British Columbia, Canada | AY248791 | FJ639545 | AY248832 | AY248816 | FJ639526 | FJ639581 |
| *Nymphalis polychloros* | NW62-2 | Sweden | AY248788 | FJ639541 | AY248829 | AY248813 | EU141502 | EU141401 |
| *Nymphalis xanthomelas* | NW84-1 | Yakutia, Russia | AY248790 | FJ639546 | AY248831 | AY248815 | FJ639527 | FJ639582 |
|  |  |  |  |  |  |  |  |  |
| Ingroup taxa |  |  |  |  |  |  |  |  |
| *Kaniska canace* | EW19-10 | Japan | FJ639396 | - | - | - | - |  |
| *Kaniska canace* | EW19-11 | Japan | AY248792 | FJ639540 | AY248833 | AY248817 | FJ639528 | FJ639583 |
| *Kaniska canace* | NW164-1 | Laos | FJ639397 | - | FJ639395 | FJ639493 | FJ639529 | FJ639584 |
| *Polygonia c-album kultukensis* | EW13-1 | Buryatia Republic, Russia | FJ639398 | - | FJ639345 | X | FJ639499 | FJ639551 |
| *Polygonia c-album hamigera* | EW13-3 | Primorskiy Krai, Russia | FJ639399 | - | FJ639350 | X | - | - |
| *Polygonia c-album imperfecta* | EW26-32 | Morocco | FJ639400 | - | - | X | - | - |
| *Polygonia c-album c-album* | NW70-3 | Sweden | AY090222 | FJ639533 | AY090154 | AY090188 | FJ639514 | FJ639569 |
| *Polygonia interposita* | NW166-6 | Kirgisia | FJ639418 | - | FJ639394 | FJ639490 | FJ639511 | FJ639566 |
| *Polygonia c-aureum* | EW13-10 | Primorskiy Krai, Russia | FJ639401 | - | FJ639346 | - | - | - |
| *Polygonia c-aureum* | EW13-12 | Amur Area, Russia | FJ639402 | - | FJ639347 | X | FJ639500 | FJ639552 |
| *Polygonia c-aureum* | NW65-8 | Japan | AY248799 | AF412754 | AF412786 | AY248824 | FJ639513 | FJ639568 |
| *Polygonia comma* | EW21-10 | North Carolina, USA | FJ639404 | - | FJ639370 | - | - | - |
| *Polygonia comma* | EW21-11 | North Carolina, USA | FJ639405 | - | FJ639371 | - | X | - |
| *Polygonia comma* | NW65-6 | Tennessee, USA | AY248794 | AF412732 | AF412781 | AY248819 | FJ639512 | FJ639567 |
| *Polygonia egea* | NW63-1 | Greece | FJ639407 | - | - | FJ639492 | - | - |
| *Polygonia egea* | NW77-15 | Greece | AY248800 | FJ639539 | AY248838 | AY248825 | FJ639520 | FJ639575 |
| *Polygonia egea* | NW120-7 | Iran | FJ639406 | - | FJ639390 | FJ639486 | FJ639507 | FJ639562 |
| *Polygonia undina* | EW30-1 | Tadjikistan | FJ639452 | - | FJ639389 | FJ639485 | FJ639505 | FJ639560 |
| *Polygonia undina* | EW33-24 | Uzbekistan | FJ639453 | - | - | - | - | - |
| *Polygonia undina* | EW43-1 | Kirgisia | FJ639454 | - | - | - | - | - |
| *Polygonia undina* | EW43-2 | Kirgisia | FJ639455 | - | - | - | - | - |
| *Polygonia faunus rusticus* | EW12-7 | British Columbia, Canada | FJ639408 | - | FJ639343 | X | FJ639498 | FJ639550 |
| *Polygonia faunus hylas* | EW19-1 | New Mexico, USA | FJ639409 | - | FJ639365 | X | X | FJ639553 |
| *Polygonia faunus silvius* | EW21-12 | Quebec, Canada | FJ639410 | - | - | - | - | - |
| *Polygonia faunus faunus* | EW21-13 | Quebec, Canada | FJ639411 | - | FJ639372 | X | X | FJ639555 |
| *Polygonia faunus rusticus* | NW74-12 | Oregon, USA | AY248798 | FJ639535 | AY248837 | AY248823 | FJ639516 | FJ639571 |
| *Polygonia g-argenteum* | NW165-2 | Mexico | FJ639412 | - | FJ639392 | FJ639488 | FJ639509 | FJ639564 |
| *Polygonia gigantea* | NW166-5 | China | FJ639413 | - | FJ639393 | FJ639489 | FJ639510 | FJ639565 |
| *Polygonia gracilis* | EW21-14 | Quebec, Canada | FJ639414 | - | - | - | - | - |
| *Polygonia gracilis* | EW22-8 | New Hampshire, USA | FJ639415 | FJ639531 | FJ639383 | FJ639484 | FJ639503 | FJ639558 |
| *Polygonia gracilis* | EW22-9 | Quebec, Canada | FJ639416 | - | FJ639384 | X | X | FJ639559 |
| *Polygonia zephyrus* | EW10-9 | Wyoming, USA | FJ639462 | - | FJ639339 | - | FJ639496 | FJ639549 |
| *Polygonia zephyrus* | EW10-10 | Wyoming, USA | FJ639457 | - | - | - | - | - |
| *Polygonia zephyrus* | EW10-11 | Wyoming, USA | FJ639458 | - | - | - | FJ639494 | FJ639547 |
| *Polygonia zephyrus* | EW10-12 | Wyoming, USA | FJ639459 | - | FJ639338 | X | FJ639495 | FJ639548 |
| *Polygonia zephyrus* | EW10-13 | Wyoming, USA | FJ639460 | - | - | - | - | - |
| *Polygonia zephyrus* | EW10-14 | Wyoming, USA | FJ639461 | - | - | - | - | - |
| *Polygonia zephyrus* | EW11-4 | Wyoming, USA | FJ639463 | - | - | - | - | - |
| *Polygonia zephyrus* | EW11-9 | Oregon, USA | FJ639464 | - | - | - | - | - |
| *Polygonia zephyrus* | EW14-10 | British Columbia, Canada | FJ639465 | - | - | - | - | - |
| *Polygonia zephyrus* | EW14-11 | British Columbia, Canada | FJ639466 | - | - | - | - | - |
| *Polygonia zephyrus* | EW14-12 | British Columbia, Canada | FJ639467 | - | - | - | - | - |
| *Polygonia zephyrus* | EW14-14 | British Columbia, Canada | FJ639468 | - | - | - | - | - |
| *Polygonia zephyrus* | EW14-15 | British Columbia, Canada | FJ639469 | - | - | - | - | - |
| *Polygonia zephyrus* | EW15-1 | British Columbia, Canada | FJ639470 | - | FJ639356 | X | X | X |
| *Polygonia zephyrus* | EW15-2 | Washington, USA | FJ639472 | - | FJ639358 | X | X | X |
| *Polygonia zephyrus* | EW15-3 | Washington, USA | X | - | - | - | - | - |
| *Polygonia zephyrus* | EW15-4 | Washington, USA | FJ639473 | - | FJ639359 | X | X | X |
| *Polygonia zephyrus* | EW15-15 | British Columbia, Canada | FJ639471 | - | - | - | - | - |
| *Polygonia zephyrus* | EW19-7 | Colorado, USA | FJ639456 | - | FJ639368 | - | - | FJ639554 |
| *Polygonia zephyrus* | EW23-18 | Alberta, Canada | X | - | FJ639385 | X | FJ639504 | - |
| *Polygonia zephyrus* | EW23-19 | Alberta, Canada | X | - | - | - | - | X |
| *Polygonia zephyrus* | NW63-11 | British Columbia, Canada | FJ639474 | - | - | - | - | - |
| *Polygonia zephyrus* | NW74-5 | Wyoming, USA | FJ639475 | FJ639536 | - | - | - | - |
| *Polygonia zephyrus* | NW74-6 | Wyoming, USA | AY248797 | - | AY248822 | AY248836 | FJ639517 | FJ639572 |
| *Polygonia haroldii* | NW112-3 | Mexico | AY788662 | FJ639532 | AY788560 | AY788800 | FJ639506 | FJ639561 |
| *Polygonia haroldii* | NW165-1 | Mexico | FJ639417 | - | FJ639391 | FJ639487 | FJ639508 | FJ639563 |
| *Polygonia interrogationis* | EW22-12 | Tennessee, USA | FJ639419 | - | - | - | - | - |
| *Polygonia interrogationis* | EW22-14 | New Hampshire, USA | FJ639420 | - | FJ639379 | - | - | ­- |
| *Polygonia interrogationis* | NW77-12 | Tennessee, USA | AY248793 | FJ639538 | AY248834 | AY248818 | FJ639519 | FJ639574 |
| *Polygonia oreas silenus* | EW14-7 | British Columbia, Canada | FJ639421 | - | FJ639353 | FJ639477 | X | X |
| *Polygonia oreas silenus* | EW14-8 | British Columbia, Canada | FJ639422 | - | FJ639354 | FJ639478 | X | X |
| *Polygonia oreas silenuis* | EW14-9 | British Columbia, Canada | FJ639423 | - | FJ639355 | FJ639479 | X | X |
| *Polygonia oreas silenus* | NW74-10 | Oregon, USA | AY788663 | FJ639534 | AY788561 | AY788801 | FJ639515 | FJ639570 |
| *Polygonia oreas silenus* | NW74-11 | Oregon, USA | FJ639424 | - | - | - | - | - |
| *Polygonia progne* | EW19-9 | Alberta, Canada | FJ639425 | - | - | - | - | - |
| *Polygonia progne* | EW21-1 | Quebec, Canada | - | - | - | FJ639480 | - | - |
| *Polygonia progne* | EW21-2 | New Hampshire, USA | FJ639426 | - | FJ639373 | FJ639481 | X | FJ639556 |
| *Polygonia progne* | EW21-3 | Virginia, USA | AY248795 | FJ639530 | FJ639374 | FJ639482 | - | - |
| *Polygonia progne* | EW21-4 | Quebec, Canada | FJ639427 | - | FJ639375 | FJ639483 | FJ639502 | FJ639557 |
| *Polygonia progne* | EW21-16 | Quebec, Canada | FJ639476 | - | - | - | - | - |
| *Polygonia progne* | EW22-15 | New Hampshire, USA | FJ639428 | - | - | - | - | - |
| *Polygonia progne* | EW22-16 | New Hampshire, USA | FJ639429 | - | - | - | - | - |
| *Polygonia progne* | EW22-17 | Quebec, Canada | FJ639430 | - | - | - | - | - |
| *Polygonia progne* | EW22-18 | Quebec, Canada | FJ639431 | - | - | - | - | - |
| *Polygonia progne* | EW22-19 | Quebec, Canada | FJ639432 | - | - | - | - | - |
| *Polygonia progne* | EW22-20 | New Hampshire, USA | FJ639433 | - | - | - | - | - |
| *Polygonia progne* | EW22-21 | New Hampshire, USA | FJ639434 | - | - | - | - | - |
| *Polygonia progne* | NW63-10 | Wisconsin, USA | FJ639435 | - | - | - | - | - |
| *Polygonia satyrus* | EW11-10 | Idaho, USA | FJ639436 | - | FJ639340 | X | FJ639497 | - |
| *Polygonia satyrus* | EW11-11 | Oregon, USA | FJ639437 | - | - | - | - | - |
| *Polygonia satyrus* | EW11-12 | Oregon, USA | FJ639438 | - | - | - | - | - |
| *Polygonia satyrus* | EW15-9 | British Columbia, Canada | FJ639445 | - | FJ639360 | X | FJ639501 | - |
| *Polygonia satyrus* | EW15-10 | British Columbia, Canada | FJ639439 | - | - | - | - | - |
| *Polygonia satyrus* | EW15-11 | British Columbia, Canada | FJ639440 | - | - | - | - | - |
| *Polygonia satyrus* | EW15-12 | British Columbia, Canada | FJ639441 | - | FJ639357 | X | X | - |
| *Polygonia satyrus* | EW15-13 | British Columbia, Canada | FJ639442 | - | - | - | - | - |
| *Polygonia satyrus* | EW15-14 | British Columbia, Canada | FJ639443 | - | - | - | - | - |
| *Polygonia satyrus* | EW15-16 | British Columbia, Canada | FJ639444 | - | - | - | - | - |
| *Polygonia satyrus* | EW16-6 | British Columbia, Canada | FJ639446 | - | - | - | - | - |
| *Polygonia satyrus* | EW16-7 | British Columbia, Canada | FJ639447 | - | - | - | - | - |
| *Polygonia satyrus* | EW23-13 | Alberta, Canada | FJ639448 | - | - | - | - | - |
| *Polygonia satyrus* | EW23-14 | Alberta, Canada | FJ639449 | - | - | - | - | - |
| *Polygonia satyrus* | NW65-9 | Washington, USA | FJ639450 | - | - | - | - | - |
| *Polygonia satyrus* | NW74-8 | Oregon, USA | FJ639451 | - | - | - | - | - |
| *Polygonia satyrus* | NW74-9 | Oregon, USA | AY248796 | FJ639537 | AY248835 | AY248821 | FJ639518 | FJ639573 |
